# A concept in flux and starved of the metaphysical: desecularizing emotion

**DOI:** 10.3389/fpsyg.2024.1373443

**Published:** 2024-05-13

**Authors:** Tamim Mobayed

**Affiliations:** Wolfson College, The University of Oxford, Oxford, United Kingdom

**Keywords:** emotion, moral emotions, Islamic psychology, philosophy of psychology, moral psychology, orientalism, secularism

## Abstract

Despite being of undisputed importance, there is no consensus on what emotions are, with the majority of research that constructs ideas about them being colored by a particular worldview. This conceptual article examines the case for conducting an Islamic analysis of emotion. It might seem a peculiar area to examine; what would be the need to analyze such a universal psychological concept through the lens of a particular religion? Three points are used to argue for this endeavor. To begin with, this article highlights the relative instability of the term; there is yet no universally agreed upon definition of what emotions are, and which human processes they contribute to. As the concept is still being defined, there is merit in engaging with the discussion, particularly given the relative absence of metaphysics from the debate. Secondly, specificities relating to emotions and how they have conceptualized are considered. This section delves into the way in which variable factors, such as culture, language, and worldview, influence how emotions manifest. The overarching point argued for here is that how emotions are experienced, and even which emotions are experienced, are shaped by factors that are not consistent across time and space. Accordingly, different worldviews will formulate different “emotional palates” and “emotional ideologies”; different groups of people will understand and engage with emotions differently. Thirdly, a broader discussion ensues pertaining to the nature of science, psychology, and their relationship with secularity. This discussion includes critique of the idea that forces such as secularism and scientific materialism have been “discovered” and are therefore value-neutral. Accordingly, understandings of emotions to have emerged from the Academy, and contemporary psychology, are value-laden. This section also looks at the origins of science in order to determine whether it is inherently atheistic or areligious, and therefore antagonistic to a religious worldview. The section also challenges the apparent secularity of psychology and key psychologists. A range of other facets pertaining to how the emotions have been conceptualized, such as their relational core, their interaction with moral faculties, and their enmeshment with what is important to individuals and groups, are also considered.

## 1 Introduction

“Western folk psychological categories [reflect] a particular, culturally specific theory of mind…Since the professional and publishing institutions of psychological science are filled with people who likely possess and implicitly employ this theory of mind, we cannot, as a field, reconsider these most basic assumptions without also questioning our experience of gravity and the solidity of the objects we interact with. But question we must, because it is distinctly possible that, for a very long time, psychological scientists have misunderstood the nature of the very phenomena that we are attempting to understand. With this challenge in mind, let us strap on our seat belts and take the plunge.” (Barrett, 2022, 903)

These are not the musings of a theologian in the seminary, or a pseudo-scientist of the fringes, but an academic who is among the most cited 1% in the world. Lisa Feldman Barrett is of the preeminent psychologists concerned with the emotions, and is a towering figure within the field. While there are those who disagree with her positioning, her ideas command respect and are certainly within the bounds of acceptability. Barrett’s call to re-examination and interrogation of the area of emotions is be to taken seriously. This interrogation includes consideration of the influence of “Western individualism…” which in her view, “…biases scientific thinking” (Barrett, 2022, 903); in an earlier publication she wrote of current psychological paradigms being part of a “cherished narrative in Western civilisation” (Barrett, 2017, 1), seemingly one that is contestable.

This level of challenge to the status quo reflects one facet of broader debate within the field of emotions, wherein the definitions and delineations are ever being negotiated. Many definitions of the emotions there are, and there are even more claims regarding what emotions do. With this deluge of theories and models already published, it is fair to ask if an Islamic treatment of the emotions and the moral emotions is superfluous. This paper attempts to address this very question; why would an Islamic treatment of the emotions be warranted, let alone needed? The first and second parts of the answer herein hones in on specific aspects of emotions and understandings of them. The third part considers wider questions about the epistemology of “secular psychology” and “Islamic psychology”, and how these considerations fuel the need for an Islamic analysis on this topic. The reality of a deluge of theories and models also supports the proposal itself; this range is revelatory of a concept, and according to significant voices, a construct, that is ill-defined and upon whom discussion and debate is ongoing. Perhaps just as importantly, one might suggest that answers to questions pertaining to what an emotion is, and what its functions are, depend on who is asking and who is answering. Pushing a little further into this area, a further point of enquiry would be to question the epistemological underpinnings of the Academy that has produced most of the contemporary knowledge that is held on the emotions; the work of those such as Wael Hallaq and Recep Şentürk will be instructional to this end (Hallaq, 2012, 2018; Şentürk, 2022). Barrett’s making salient the extent of Western influence on psychology, and understandings of emotions, should not go amiss (Barrett, 2017, 2022).

While the boundaries are contested, the importance of the emotions are not. It has been said of them that they, “are what make life interesting, and what makes us feel important. From this point of view, they are the most valuable element in human existence” (Stanghellini and Rosfort, 2013; Scarantino and de Sousa, 2021). Others have focused on the way in which they “change the way that we think, feel, and behave in powerful ways…the changes wrought by emotion have real-world implications for whether or not we succeed in attaining our goals, for our relationships with others, and for our well-being and life satisfaction” (Lench and Carpenter, 2018,1–2). Emotions are evidently important.

The emotions have been conceptualized in more ways than there are branches of knowledge concerned with them. Crucially, there is no one agreed upon definition of what an emotion is and is not, with Barrett citing Lakoff who wrote, “everyone agrees that emotions exist but no one can agree on their definition” (Barrett and Russell, 2015, 4). Such is the debate around them that those such as Niedenthal and Brauer (2012) question whether emotion as a concept can be studied at all, seemingly due the variability of the definitions of the term. Dukes et al. (2021) effectively counter such questioning with a timely reminder that many other complex concepts have proven beyond a one, universal definition yet are soundly researched. The significance of emotions to the life of the individual, and the life of groups, as well as the apparent instability of the way(s) in which they are conceptualized, make them ripe for analysis and discussion.

To better understand the emotions, scholars have noted the need to integrate knowledge “from the cultural to the behavioral, psychological, experimental, physiological, and molecular” (Coan and Allen, 2008, 8), while others instead mention the “biological and neurological, behavioral, cultural, structural, and situational” (Turner, 2009, 341). Others still have suggested ethology, evolutionary biology, paleto-anthropology, philosophy, neuro-biology, cognitive science, as well as psychology (Adolphs and Andler, 2018). The absence of theology or any related branch of knowledge is both expected and telling; this absence is indicative of what Taylor terms our “secular age,” and itself suggests the need for projects such as the one at hand.

Interdisciplinary sprawl is compounded by significant intra-disciplinary differences too. The range of functions that have been attributed to the emotions reflects both their sizeable and varied purpose, and the complexity of their nature. This range also demonstrates their importance to different processes. Emotions have been theorised as being central to motivation (Prinz and Nichols, 2010), appraisal (Scarantino and de Sousa, 2021), interpersonal communication (Parkinson, 2020), intrapersonal communication (Reisenzein, 2007), readying an individual to react (Barrett, 2012), appraising “performance” post-reaction (Lefebvre and Krettenauer, 2019), commandeering key faculties to allow for a comprehensive reaction (Sander and Scherer, 2009), and more. It is believed that emotions have a specific relationship with things that have meaning to the individual, or are critical for their survival, both in the most basic sense, e.g., surviving from an impending physical threat (McKenzie et al., 2019), and to more abstract factors to thriving, e.g., aligning to meet one’s long term goals (Nussbaum, 2001). Phenomenologists go as far as theorising that emotions link the body to the world to create meaning, and assisting an individual’s grasping of meaning of worldly objects (Stanghellini and Rosfort, 2013). They have been deemed as manifesting as feelings, thoughts, behaviors, and physiological responses. Some believe emotions to be universal and essentially biological (Ekman, 1993); others go to the opposite end of that spectrum and believe them to be wholly socially and culturally constructed (Barrett and Russell, 2015).

Driving the study at hand, the ideologies dominating the contexts within which emotions have been studied also plays a factor in the knowledge derived about them. Plato’s ideas have long dominated thinking about the emotions, and knowledge as a whole (Giner-Sorolla, 2013). While approaches varied, Hellenic philosophers set a general tone of suspicion towards the emotions which long carried through into most of the two millennia that succeeded theirs. It was not until the Renaissance that emotions began being viewed in a positive light (Williams, 1998), certainly, within the Western world. The influence of Hellenic philosophy on Islamic thought is of course of significance. According to Williams’s (1998) telling, Plato may have been the first mover in this trend of suspicion towards emotions, but it is the Enlightenment that deals it a significant and lasting boost. Williams’ assertion that the Enlightenment is significant in subduing the value of emotions is relevant to discussions about religious approaches to emotions, given the Enlightenment’s largely antagonistic relationship with religion. In the vein of antagonistic relationships with religion, Freud obliges and maintains this tradition, with psychology eventually moving past its psychodynamic birth, into behaviorism, then cognitivism, until finally, something of an age of affectivism sprouts (Haidt, 2001; Dukes et al., 2021). This is a brief telling of the history of Western psychology, though undoubtedly the “Muslim world” has been influenced by it, as shall be discussed (Hallaq, 2012, 2018; Şentürk, 2022). The idea that the context from which our current body of work on emotions emerged is part of the story of what we know (or think we know) about emotions is worth keeping in mind, and is one driver of this study. Illustrating this idea is Taylor’s assertion that secularism was not discovered, but rather, was constructed (Taylor, 2007).

### 1.1 The malleability of emotional manifestation

If hard, biological, universalist theories—that argue that emotions are exclusively biophysiological and are experienced universally by all in nearly the same manner—are to be believed, the importance of an Islamic conceptualization of the emotions becomes less important, if important at all. In this telling, emotions are impervious to individual and cultural variation, or, individual and cultural variation play insignificant roles in their function and expression. Significant figures in the field of emotions, such as Ekman, do indeed argue that emotions are largely biological and universal (Ekman, 1993). The counter position to emotional universalism is well described by Mesquita et al. (2016) who note that emotions are not something that humans simply “have,” but rather, that emotions are something people “do,” such is the role that construction plays in emotional experience. While a full examination of this debate falls outside the scope of this paper, it seems more likely that processes such as individual appraisal, and cultural variation, play a role in how emotions are experienced (Lazarus, 1993; Barrett, 2017). Rather than debating whether the individual and their culture play any role in how emotions are manifest and understood, the livelier debate centres around just how much of an influence these factors play. One compromise from amidst emotional universalism is offered in the characterisation of the emotions as being universal yet exhibiting different dialects (Scarantino and de Sousa, 2021). Another route to reconciliation is found in differentiating between basic emotions (e.g., fear and sadness), and complex emotions (e.g., shame and gratitude); universalists tend to focus on basic emotions.

James’ seminal paper from more than a century ago made the claim that emotions are essentially a feeling derived from (1) a stimulus in the environment which leads to (2) a physiological response and results in (3) an individual’s interpretation of that physiological response (James, 1884). What became commonly known as the James-Lange theory places great significance on the individual’s perception and interpretation. Since then, different theories have advanced, deepened, and added nuances to the role that an individual’s interpretation of what is going on plays on the experience of emotions.

Lazarus and Folkman’s theory of stress is a useful example of this, with it placing a great deal of significance on the way individuals appraise a potentially stressful stimuli (Lazarus and Folkman, 1984). Lazarus’ later theory goes further, postulating that individuals who appraise a potential stressor as a challenge, and something that might be beneficial and conducive to growth can experience stress in a different way, with it being experienced as eustress (Lazarus, 1993). Appraisalists argue that appraisal plays an important role in the way emotions are experienced, with the process of appraising depending on cognitions, which are shaped by factors such as personality and culture. Stimuli might be objective, but their interpretation and assessment is very much subjective.

Barrett’s work is apparently most popular of those making the claim that emotions are in large part constructions (Barrett, 2012, 2017, 2022; Barrett and Russell, 2015). Barrett’s (2022) work develops this theme within the context of her belief that much of the world is existing within people’s minds:

“Goals, value, affect, and other mental features are not properties that exist in the world or the body. They are features that exist only in a brain that creates these relational ensembles.” (Barrett, 2022, 906)

The idea that reality primarily exists in minds is seen by Taylor as being among the forces that foment and give rise to secularism; he relates it in particular to another force in atomisation (Taylor, 2007).

As well as giving significance to individual interpretation, Barrett’s work makes salient the social and cultural element of this. Her determination of emotions as “ontologically subjective categories” (Barrett, 2012), put the individual, and the individual as a member of a community, at the center of the process by which emotions are understood and experienced. Barrett (2012) writes on the significance of group-level understanding of emotions; according to her, emotions are only as effective, and as real, as a group’s understanding of them. Without a shared understanding, emotions can exist, but are arguably devoid of significance and meaning. Illustrating this point well, Barrett leans on the work of philosopher John Searle’s reflections about what determines whether a plant is a flower or a weed (Searle, 1995). The meaning of this is created and shared within a group, and allows them to communicate and influence one another; collective intentionality and understanding is key.

Barrett articulates:

“Humans create ontologically subjective categories to serve functions that help constitute social life…such functions are the glue that holds a human society together. If emotion categories are ontologically subjective categories, then they can be thought of as collective tools that allow members of the same culture (and even different cultures, depending on the categories, of course) to represent and shape the social meaning of physical events.” (Barrett, 2012, 419)

Emotions are experienced within a social reality, with the members of the community playing a role in constructing what this meaning is. The significance of this point is deepened still when one reflects on the comparatively concrete nature of plants, as opposed to the immaterial constitution of emotions. Groups construct different meanings about emotions, and develop different vocabulary to reflect them, which in turn impacts how they are experienced. If Barrett’s thesis on the role that groups play in constructing meanings about emotions is even partially true, it serves to support the proposal at hand. The idea of boundaried groups and intragroup construction of meaning invites consideration of the Islamic concept of ummah. Stemming from this point is another of significance– all terms are informed by a worldview, with the secular materialistic worldview that dominates the Academy today being as much of a construction as any other; Taylor’s point about secularism’s construction rather than discovery again comes to mind (Taylor, 2007).

Taylor’s writings on the significance of language argue that it is particularly in the world of feelings and other abstract concepts that language becomes critical. Contrasting the neatness of using language to describe “things”, and more universal emotions like sadness and happiness, Taylor notes the significant role played by a culture’s language in facilitating emotional experience:

“But how do we, either individually or as a culture, go beyond these obvious, basic cases, and find more refined and subtle terms for how we feel: ‘uneasy’, ‘troubled’, ‘serene’, ‘alienated’? How do we learn to describe our world as full of meaning, or flattened, deprived of meaning? Unlike the basic cases, these feelings/meaning arise in certain cultures and not others, and they are connected through skeins of meaning to a whole host of other discriminations which belong to this culture: its virtues, values, morals, sense of beauty, sense of fullness, its understandings of shame, and (where this is important) guilt….” (Taylor, 2016, 187)

Taylor describes the motivational force that is generated by an individual’s better understanding themselves through the words they know and apply, writing on it bringing clarity to confusion, and clarifying a “sense of what really matters.” Laden within his ideas here is the catalysation of a sense of catharsis that can emerge from better understanding oneself, with language playing a central role in this. With this in mind, ideas such as those of Lazhar gain weight (Lazhar, 2023). Lazhar writes on the Islamic worldview, and more specifically, the Quran as having, “insufflated a new semantic life to the Arabic vocabulary…evolving in the context of a distinct worldview and value system, the word [in this case, salat/ritual prayer] now denotes a new reality in which etymology and custom only play a secondary role” (Lazhar, 2023, 162). Taylor describes the importance of language to knowledge and self-knowledge, while Lazhar describes the Islamic worldview as having reanimated the language used by its adherents. The role that language plays in shaping realities evokes ideas such as the Sapir-Whorf hypothesis (Scholz et al., 2024). This thesis posits that language plays a direct and directive role in influencing thought, to the extent that different languages uniquely influence their speakers.

Coulter adds to this position with his writings on the social construction of emotions (Coulter, 1979). Using the example of shame, he highlights the strength of a social aspect to its manifestation by way of the fact that shame is rooted in right and wrong, and the violation of societal standards:

“No matter how much a dog may cringe with its tail between its legs when caught in the act of dragging its bone across the rug, it does not feel guilt or shame. Whatever it is that the dog feels (fear?) the ascription of categories such as guilt, shame, or remorse, apply only by analogical extension….” (Coulter, 1979, 132–133)

Coulter’s specific mention of the moral emotions here calls to mind an idea from Taylor and the related idea of the social construction of morality:

“We are constantly losing from sight…that being a self is inseparable from existing in a space of moral issues…the real difficult thing is distinguishing the human universals from the historical constellations and not eliding the second into the first so that our particular way seems somehow inescapable for humans as such, as we are always tempted to do.” (Taylor, 1989, 112)

If emotions are partly socially constructed, and morality is partly socially constructed, different groups would likely benefit from analysing psychological phenomena through the lens of their own worldview. In fact, those such as Tagney have deemed moral emotions such as shame as psychological “moral barometers” (Tangney et al., 2007). As well as construction, they serve a distinct moral function. Different worldviews will surely produce different barometers based on their different needs.

The relational aspect of emotions has been well established, with many of the proposed functions of emotions relating to others, by way of communicating with them, expressing something to them, influencing what they feel so as to influence what they think and what they do, and so on. While an individual’s relationship with God is largely absent from the Western Secular academy, it remains at the forefront of the Islamic creed; it could even be argued that no other relationship matters in a comparative sense. The Islamic conception of the importance of god-consciousness is unrelenting. Conceptualizing what emotions are, while giving them a strongly relational focus, sets up emotions in a way so as to serve human relationships. Inserting, or re-inserting, as colossal a figure as The Almighty within that plane would necessarily change the dynamics of how emotions are understood, and what functions they are believed to conduct. Within a web of relational understanding, God’s gravitational pull would markedly impact on the entire structure.

Which relationships are important, and what constitutes morality, are concerns that relate to worldviews. Further to the idea that a comprehensive worldview would want to involve itself in the fundamental workings of how its harbourers navigate their world, another suggested function of emotions becomes relevant. According to a number of scholars, emotions aid individuals in focusing to what is important to them, including concerns of one’s hopes and values (Nussbaum, 2001; Lemmings and Brooks, 2014; Vallerand, 2015). Relatedly, González (2016) wrote on the emotions being directed at what one care’s about. Dukes et al. (2021) furthered this point by claiming that emotions do not only play a role in focusing on what is important, but they also aid the individual in filtering out what is not important. Emotions concern themselves with what is important and what is not; worldviews, and especially religious worldviews, also concern themselves with these areas. Relying on a secular psychological approach to the emotions, or even simply approaches that are not cognisant of the metaphysics of Islam, seems ill-fitting.

Another important idea comes from Hochschild and the significance of “emotion ideologies,” wherein groups determine rules pertaining to emotions; what should be felt, what should be displayed, what emotion labels are valid, and how they should be talked about (Bellocchi and Turner, 2019). Apparent here is a high level of both prescriptivity and construction. Rather than touching on the way emotions are experienced through primarily a psychological lens, the idea of emotion ideologies instead highlights the different rules groups have for emotions and their expression. Much of the cross-cultural work on emotions highlights that different emotion ideologies already exist amongst most groups (Mesquita et al., 2016). What is less forthcoming is an attempt to delve into this topic in a structured way, drawing from Islamic sources. The three preceding points tie into each other; which relationships are important, what kind of things are important to the individual, and what their emotional ideologies might be, all feed into the core facets of a worldview. Different worldviews will likely have different answers to each.

As well as being experienced differently, and being composed of different dialects (Scarantino and de Sousa, 2021), it is likely that the Islamic palate for emotions will differ from those of other worldviews. Going directly to the heart of the Islamic worldview—the Quran—and certain examples are forthcoming:

ثَانِىَ ٱثْنَيْنِ

إِذْ هُمَا فِى ٱلْغَار

إِذْ يَقُولُ لِصَـٰحِبِهِۦ

ۖلَا تَحْزَنْ إِنَّ ٱللَّهَ مَعَنَا

فَأَنزَلَ ٱللَّهُ سَكِينَتَهُۥ عَلَيْه

… and he was only one of two.

While they both were in the cave,

he reassured his companion,

“Do not worry; Allah is certainly with us.”

So Allah sent down His serenity upon him (Quran 9:40).

There is in this verse an indication of serenity/سكينته being an emotional state that not only exists, but is directly sent by God to the very heart of an individual as an aid in the face of another emotion, grief/sadness/حزن. It is not the only verse that speaks of serenity being sent to the hearts of humans (e.g., Quran 48:4, 48:26).

A further verse that suggests there is much to be mined and garnered from Islamic sources vis-à-vis emotions and an Islamic approach to them is found in chapter 53:

وَأَنَّهُۥ هُوَ أَضْحَكَ وَأَبْكَىٰ

And that it is He who makes [one] laugh and weep (Quran 53:43).

Here appears to be again a direct relationship drawn between Divine Power and Action, and an individual’s emotions. While the first example could be dismissed as applicable to certain unique classes of people (i.e., prophets and their companions), this verse appears to be both general and universal.

The Quran introduced itself to the world with the instruction to “read”, followed closely with mention of the sacredness of the act of writing – two behaviours that arguably stoke cerebral chords more than emotive ones. As readers progress into the second, and lengthiest, chapter, the steady rhythm of the verses yield a repeated message that is emotionally orientated and designed to comfort and guide; no fear and no grief will be upon those who believe and act morally. Words to this effect are repeated multiple times throughout the book, and five times within that second chapter alone. The Quran depicts the story of Moses, as he embarks on his journey, during which his fears are first comforted by God directly – “do not fear” - through verbal reassurance, as well as the instilling of self-efficacy. Eventually, his conquering of his fears allows him to become in turn comforter to the fears of his flock. Implicit yet salient within the Quran’s transmission of the speech of God, dealing directly with Moses’ emotion, is a significant validation of the overall importance of emotions, and the value in attending to them. Within the Prophet Muhammad’s life, the tenderest moments serve as opportunities to break from Pre-Islamic emotional norms, and develop Islam’s own emotional ideology. These include the heart-wrenching final moments of his dying infant son, Ibrahim, or the Prophet’s instructions to a man who proclaims that he never kisses his children. These parables and the instructions within them directly challenged the emotional ideologies of the age. The Prophet’s final moments with his son and his experiential instruction to allow poignant sadness to emerge, contrast with the Quranic Jacob, his searing pain, and his almost mystical declaration of only sharing his emotional pains with his Lord. Even in the moment of triumph, the Prophet Muhammad again carves a new emotional path for this followers; humility and gratitude mark the order of the day, as riding on camelback, he slumps in submission to his Maker, drawing yet another alternate emotional path from the pride and vengefulness that would have characterised the triumphs of the monarchs of neighboring Persia and Rome. The Islamic tradition is loaded with emotive energy and instruction, yet it has not been rendered impervious to the influence of other worldviews and their respective, and all too often imposing, emotional ideologies. More research is needed, but the promise is apparent.

Another relevant issue pertains to the aversion of key thinkers to metaphysical and religious explanations. Darwin’s own aversion to seemingly more likely explanations because they would point towards a Designer should be noted. Parkinson (2005) cites the work of Fridlund in his determination that Darwin wanted to resist conceding that facial expressions were tied to emotions, because that would imply the purposeful design of a Designer. Furthermore, scholars who are not averse to religion should then wonder what other ideas have been coloured by such goals, and what steps should be taken to address such issues; conscious and unconscious biases have undoubtedly played their respective and often imposing roles.

While the jury remains out on the precise role that emotions play in moral decision making, whether they are essentially the heart of moral behavior, or they merely play a supporting role (and can often in fact be absent), most scholars agree that they are present somewhere within the equation. Even if the extreme objectivism camp is correct, emotions play a significant role in moral decision making, albeit a wholly negative one. Whether they are contributors to moral decision making or the bane of morality, they are relevant, and so are important to any worldview that concerns itself with morality.

The goal of most religions’ attempt to drive the whole moral life of the individual, and so the relationship between religion and emotion is one of significance. More broadly, the jury also remains out on what exactly constitutes an emotion (Barrett and Russell, 2015). With the concept itself under scrutiny by a few, and being wrestled over by many, it would seem apt for believers in a worldview such as the Islamic to engage and contribute to the discussion. As well as challenges to the very essence of the term, Muslim scholars would be wise to note the apparent absence of God from the vast majority of conceptualizations of what emotions are and what their function is. This is surely something believers in the Islamic worldview would strive to remedy, perhaps doing the reverse of what Darwin set out to do, by *a priori* removing a Designer from his conception; the starting point for Muslims on every significant truth-seeking endeavour is He/ هو and according to the Islamic conception of Him, His gravitational pull is insuperable.

### 1.2 Psychology, science, and secularity

Moving a step back from specifically looking at the emotions, broader considerations pertaining to psychology and science also contain evidence in support of the argument of this paper. Staying in the vein of the influence of anti-religious thinkers on this area of study, Ekman (2009) cites Darwin as a key influence on his theories, while Haidt cites Richard Dawkins’ Selfish Gene (Dawkins, 1976) as a significant reading in his intellectual development. Granted, Haidt also criticises certain aspects of Dawkins thinking, especially in relation to arguments made by Wilson (2002). While these key figures within the field being influenced by such significant anti-religious thinkers is not in itself a reason to dismiss any of their ideas outright, it does hold a place within wider considerations of the field. This particular line of critique engages with a principle readily found in the humanities but is typically disregarded within the sciences; the ideology of the individual influences their craft. The claim that science is objective is increasingly challenged, especially in light of the rise of whole disciplines of psychology dedicated to the study and utilisation of human biases (Thaler and Sunstein, 2008). On one side of this coin is the argument that those who harbour distinctly anti-religious worldviews play important roles in the published literature. The other side of this coin, and more positively angled, is the finding that other religions have also claimed a stake in conceptualizations of contemporary scientific psychology.

With proximity to the discussion at hand, Shweder et al.’s (1997) CAD triad hypothesis (Community, Autonomy, Divinity) of moral psychology openly cites the ideas of Hinduism in its construction, casting these as a viable alternative to contemporary American moral values. While this might be an exercise in descriptivism, the tone taken by Schweder and his colleagues at times is arguably more prescriptive than might be expected from within the secular Academy. A more distant but still pertinent example exists in the form of Gilbert’s Compassion Focused Therapy (Gilbert, 2009). Gilbert’s adaptation of cognitive behavioral therapy integrates key concepts from the Buddhist religion, again, openly and unabashedly (and rightly so!). Another instantiation of this argument is a marriage of two key figures, one from the world of emotions, the other from the world of religions. Lama et al. (2008) combines the thinking and worldviews of the Daila Lama (spiritual leader of Tibetan Buddhism) and pioneer of the psychological study of emotions Paul Ekman, in their attempt to overcome the obstacles to psychological balance and compassion, through emotional awareness. The fourth example is both controversial but also supported by a growing body of evidence. Freud, who has already been termed by some as the “godfather of modernity,” was in fact according to some, deeply influenced by Jewish mysticism in the form of Kabbalah (Alexander and Bakan, 1960), as well as his Hassidic roots (Berke, 2015). Far from being merely a cultural colouring, scholars go as far as suggesting central aspects of his psychodynamic theory, such as free association, are rooted in Kabbalist practices. Other examples of religion’s blending within psychology exist too, such as Freud’s contemporary, Carl Jung and his engagement with Christianity (Jung, 2010), while he was also comfortable to borrow from Hinduism and its practice of kundalini yoga (Jung Carl et al., 2020). There might be merit in examining whether certain religions are seen as more malleable and unthreatening so as to borrow from for the Western Academy (e.g., Hinduism, Buddhism, Judaism, and Christianity) while others are seen as more threatening (e.g., Islam). Discussions around the religiosity of other pioneers in Western psychology also exist, such as BF Skinner (Toates, 2009; Schlinger, 2011) and Carl Rogers (Fuller, 1982). Josephson-Storm extends this claim to a raft of thinkers, mostly outside of the field of psychology, but hugely influential in the development of knowledge and shaping of the contemporary world, all of whom are typically believed to be secular if not atheistic (Josephson-Storm, 2017). Josephson-Storm’s central thesis is that many of the thinkers who contributed significantly to the development of knowledge in the modern world are not as secular as is commonly believed.

The idea that psychology and psychiatry are effectively replacements for religion is held by some (Fuller, 1982; Jung, 1985). In the mid 20th century, Jung wrote:

“The wave of interest in psychology which at present is sweeping over the Protestant countries of Europe is far from receding. It is coincident with the general exodus from the Church… ‘Nowadays people go to the psychotherapist rather than to the clergyman’.” (Jung, 1985, 31)

The apparent negatively correlated relationship between religion and psychology might seem understandable, but warrants further and fuller investigation. As well as identifying the growing discontent with the Church, Jung correctly prophesised the onset of prevalence of psychological disturbance among the “most developed” world. He warned that dominant models of psychology, such as that of his peers, Freud and Alfred Adler, were ill-equipped to handle this psycho-spiritual malaise, believing them to be “hostile to spiritual values” and as being “psychology without the psyche” (Jung, 2007, 31). A leitmotif from Jung’s work is of the importance of psychology being spiritually attentive, with Jung not being shy to borrow from both Western and eastern religious traditions to this end; notably he also attempted an exegesis of the famous story of Moses and Al-Khidir from the Quran (Jung Carl et al., 2020). Most significant here is the epistemological rooting of Western psychology as a replacement for religion; there is an argument that this narrative does perhaps hold truth, however, it is relevant only to contemporary Western psychology, and not other psychologies.

As well as the need to add the Islamic, there is also a broader and less exclusively Islamic-specific argument on the merits of reintegrating the heart into psychology. While this is undoubtedly an Islamic endeavour, given the significance afforded to the heart within the Islamic psychological worldview, more specifically within the Quran, and within the work of classic Islamic scholars who approached psychology – what Moosa (2005) eloquently terms “pectoral psychology” – it is not only within the scope and interest of Muslims. A Prophetic injunction to “consult your heart” when faced with a moral decision highlights the central role that the organ plays within Islamic psychology (al-Khatib, 2022). Steinbock’s (2014) text on the moral emotions is tellingly subtitled “reclaiming evidence of the heart.” This might be seen as a station along the path to the “re-enchantment” of psychology, and science more broadly, perhaps a short few steps from the current “rise of affectivism” (Dukes et al., 2021). Taylor’s (2007) telling of the process of localisation that fuelled secularism, and more specifically, of the localising of the individual and much of reality to human minds, is worth keeping in mind. Cartesian duality marked a significant portion of this process, with the heart – once seen as of the critical components of the human – being relegated to an inert physiological organ. This relegation is in direct conflict with the Quranic worldview, and its regular, indeed dominant, addressing of individuals and their personhood as being so intimately related to their hearts. While the heart is apparently dominant, the brain is also given importance through Quranic dialogue with the عقل or intellect; tellingly, when the Quran wants to convey God’s personal intimacy to each and every human being, He tells His readers that is closer to them than their jugular vein, i.e., the connective organ between the heart and much of the head. Reintegration of the heart, and the re-enchantment of psychology, would likely entail characteristics such as a move away from scientism, a greater openness to metaphysics, and the soul/spirit, as well as literal attention to the physical heart and a redrawing of boundaries to include it within the practice of psychology. Islamic integration of the heart would also include considerations of Islamic conceptions of the human (Rothman and Coyle, 2018), as well more controversial aspects like cognisance of the devil. Challenges to Cartesian dualism are also relevant, with arguments against it increasing in volume and potency (Aungle and Langer, 2023).

There is of course a prevailing view that science is inherently disenchanted because it is fundamentally disenchanting. Stanley (2015) proposed a radically different telling. By juxtaposing the position of two Victorian scientists—one being “Darwin’s Bulldog,” Thomas Huxley,^1^ and the other being devoted Christian, James Maxwell, Stanley sheds light on the struggle for the heart of science. At the center of this battle was theism and a secularism that can be argued to be a precursor for atheistic thinking. While the details of this struggle are fascinating and relevant to all scientists—especially scientific theists—the most significant aspects to this paper are twofold. Firstly, Stanley’s claim that the roots of Western science are firmly within theistic thought; one such example he cites is the idea of the uniformity of the universe being indicative of a Devine Designer and Caretaker. Secondly, his framing of the difference between methodological naturalists, and metaphysical naturalists. The latter poses a problem for theists, while the former poses no such problem at all. Perhaps most contentiously, Stanley claims that it was the specific goal of the metaphysical naturalists to rewrite the narrative of science within the education system so as to frame science as being inherently areligious or anti-religious. While a more precise threading of this argument falls outside the remit of this study, the idea that science is not inherently anti-religious is of relevance, while the idea that science and theism are deeply related, historically if not typically within the contemporary, is relevant too. Indeed, methodological naturalism is arguably what Islamic scientists have been doing for more than a millennium. Approaching the emotions with an Islamic lens would be a rekindling of this Islamic scientific psychological spirit; in light of Stanley’s writings, the call of this paper is then less of an argument for a new approach, and more of a call to return to the roots of science.

Taylor’s work on secularism can also be positioned to support the case for an Islamic approach. Knowledge, science, and psychology are all not produced in vacuums, and the context of their production plays a fundamental role in how they are conceptualized. Taylor makes an incisive and debatable point, challenging both secularists and Platonists, but perhaps all knowledge producers too. Secularism was framed as though it were discovered and not constructed, while in classical times, Plato believed himself to be finding reason and not making it; Taylor challenges both lines of thinking (Taylor, 2007). He reinforces this position by positing that morality and ideas of personhood are ever fleeting, and are very much bound to their time periods.

“The real difficult thing is distinguishing the human universals from the historical constellations and not eliding the second into the first so that our particular way seems somehow inescapable for humans as such, as we are always tempted to do…our modern notion of the self is just as much a historically local self-interpretation which would also be opaque and perplexing to outsiders.” (Taylor, 1989, 112–113)

Going deeper into the notion of time itself, Taylor pays specific energy to the idea of the secularisation of time, wherein it is reduced to its horizontal and mechanistic forms. According to this telling, time itself, and more convincingly, the way it is experienced, is impacted by the worldview of its adherents. Such is the depth of the influence of ideologies on reality. An illuminating recent study exhibited the influence of thought on physical reality to the extent that perceptions of time were reported to have influenced physical healing (Aungle and Langer, 2023). While the influence of cognition on perception has long been reported, this study’s extension of the power of belief into the physical is noteworthy. More still can be spoken about the “social imaginary” and the “background,” as determining the contexts within which humans are living. In short, psychology produced within secularism will be different to psychology produced within another ideology.

Support for the idea of an alternate treatment of this area comes by way of Hallaq’s (2012) critique of the modern academy as a whole. According to Hallaq, educational centres are not neutral spaces by any means, rather, they are but one aspect of state institutions that are set on inculcating specific values that serve the state, as well as producing a narrow band of knowledge that sustains the state. The produced “homo modernus” is not the liberated human as claimed by that narrative, but rather a citizen efficiently inculcated with “state interests, state priorities, state programs…and state ‘problem-solving’ ideology” (Hallaq, 2012, 77). Hallaq’s surgical critique amplifies ideas mentioned about the context from which psychology emerges, and the ways in which this renders it unconducive to Islamically sensitive knowledge. A further and more troubling point of caution emerges at this juncture; has the project at hand sufficiently addressed, or even at least recognised, the extent to which the researcher and their ideas are coloured by the very systems that Hallaq warns of, given their emergence from such modern centres of education.

Şentürk’s work extends Hallaq on this point; rather than merely critiquing the academy from which they came, societal sciences as a whole are reflective of a value-laden paradigm (Şentürk, 2022). In Şentürk’s telling, “Islamicising” psychology, or any other social science, does not adhere to the holistic Islamic paradigm. Relying on Kuhn and Ibn Khaldun, Şentürk argues that approaches to human and social problems are fundamentally moulded by the civilisations from which they emerge. Accordingly, relying on a modern, Western branch of knowledge and attempting to “Islamicise” it risks descending into an act of optics and tokenism. A true return to the organically Islamic would involve engaging with psychology from within the Islamic paradigm by way of fiqh^2^, but not a fiqh that has been reduced into merely jurisprudence. Accordingly, a truly Islamic conception of the emotions would not be a mere Islamic dressing of a secular concept.

Speaking more to Şentürk’s writings, Islamic psychology is an active rejection of the position that religion and psychology are mutually exclusive, or even separable (Şentürk, 2022). Underlying this is the rejection of intellectual secularism as a whole. The practice of religious psychology, or Islamic psychology, would not be as new as it might seem, but rather, would be a rekindling of sorts. Classical Islamic scholars were readily blending knowledge of their ages, be it Hellenic or otherwise, with Islamic insights, to formulate improved theses. The most famous of these examples in relation to psychology is that of Abu Zayd al-Balkhi, a 9th century polymath. His text, Sustenance for Bodies and Souls, is replete with psychological advice so incisive that it has been compared to and found to be aligned with the DSM-5 description of phobias (Awaad and Ali, 2016). Beyond phobias, that work was laden with psychological insights that have stood the test of time, including the importance of cognitive training, socialising, and mental hygiene (Badri, 2013). Explanations as to the contributive factors to the apparent decline of Islamic psychology, evidently a reality in classical times, can be found in the work of Hallaq and warrant discussions in their own right (Hallaq, 2012, 2018). The modern field of Islamic psychology has been on a path to revive this path of knowledge. The recent work of Karen Bauer has already provided significant insights into the role emotions play within Islam, and the life of Muslims (Bauer, 2017, 2019), with other scholars also having contributed to this burgeoning field; Katz (2014) and El Shamsy (2015) in relation to the moral emotion of shame, and Lumbard (2021) for the moral emotion of gratitude. The work of Keshavarzi and Keshavarzi, and their treatment of emotions through the prism of Islamic clinical psychology is also noteworthy (Keshavarzi et al., 2020).

## 2 Conclusion

The emotions are still being understood, to the extent that a universal definition remains elusive. Given that the discussion is ongoing, and there is dearth of voices within it that are metaphysically attuned, driving a stronger religious presence within the discussion carries merit.

Setting aside deservingness of a place at the discussion, and even abandoning quests for objective truth, Muslims as an in-group, with a unique set of values and norms, would be better served by an approach to the emotions that is cognisant of their worldview. Going a step beyond, Muslims as a diverse series of in-groups likely require a number of approaches to the emotions. Emotion ideologies devised with Islam and Muslims specifically in mind appears an appropriate engagement. Relatedly, a growing number of studies support the influence of factors such as culture on emotional experience, while emotions appear to change across time too. Contemporary approaches to the emotions did not germinate in a vacuum, and have not been discovered as in the case of an objective reality, but rather, appear to have been constructed. Emotions as we have largely come to understand them from within the Academy have not been developed in a neutral way. The definitions devised, and functions proposed, are as ideologically laden as an Islamic conception of them would be. Underlying any idea about emotions, or moral emotions, or morality, or the individual, are value-laden positions that are changeable and worthy of critique. Psychology as a contemporary, modern, branch of science, is born of the Enlightenment, and is fruit of it. This manifestation of psychology is but one of many psychologies; a truly “Islamic psychology” would likely not be a mere dressing of the Enlightenment’s psychology in an Islamic coat.

A number of options seem apparent in an attempt to devise an Islamic conception of the emotions. Rather than devising an entirely independent theory (the “Islamic Psychology Approach”), they could shape conceptions of emotions to suit their needs (the “Islamic Filter Approach”); or perhaps at the very least, sifting through the theories and models that have already been proposed and determining which are in most alignment with their worldview (the “Islamic Comparison Approach”) (Kaplick and Skinner, 2017). If they are being a little braver, they would play a pioneering role in the advancement of this area of study as a whole, rather than merely its Islamic derivative. Certainly, Muslim psychologists and Islamic psychologists would see value in conceptualizing the emotions in a way that is sensitive to, or perhaps even reflects, the Islamic conception of morality and conception of the human.

The apparent malleability of emotions and emotional experiences mean that the individual, the society they are in, and the culture which colours it, influence these experiences. As a commanding and dynamic worldview, with a distinct set of moral values, and a belief in a Divine Revelation that has been preserved and is accessible today, it is natural to conclude that Muslims be interested in devising their own Islamic conceptions, theories, and approaches to the emotions, and especially the moral emotions. This drive from within the worldview is compounded by the fact psychology is still in its relative infancy, with the instability and ongoing discussions around the concept of emotion itself continuing; much remains to be settled, the concrete has by no means hardened, and Islamic scholars should be claiming their place at the proverbial table. This need not be necessary an act of divorce between mainstream contemporary psychology and Islamic psychology; examples of the influence of other religions upon mainstream psychology, and towering psychologists, are ample. The healthy development of Islamic psychology, and more specifically, an Islamic analysis of the emotions, can be an important addition and enrichment to psychology as a whole, as well as a progressive move towards enriching and deepening the emotional lives of Muslims.

## Author contributions

TM: Conceptualization, Formal analysis, Project administration, Writing – original draft, Writing – review & editing.

## Footnotes

^1^Grandfather to not one but two significant thinkers of the 20th century, Aldous and Julian Huxley.

^2^Fiqh is commonly translated as Islamic jurisprudence, and seen as Islamic law, however, those such as Șentürk argue that it is in fact broader and more multifaceted than these now narrowed definitions.
